# Multi-appearance segmentation and extended 0-1 programming for dense small object tracking

**DOI:** 10.1371/journal.pone.0206168

**Published:** 2018-10-31

**Authors:** Longtao Chen, Mingwu Ren

**Affiliations:** School of Computer Science and Engineering, Nanjing University of Science and Technology(NUST), Nanjing, Jiangsu, China; University of Science and Technology Beijing, CHINA

## Abstract

Aiming to address dense small object tracking, we propose an image-to-trajectory framework including tracking and detection, where Track-Oriented Multiple Hypothesis Tracking(TOMHT) is revised for tracking. Unlike common cases of multi-object tracking, merged detections and the greater number of objects make dense small object tracking a more challenging problem. Firstly, we handle frequent merged detections through the aspects of detection and hypothesis selection. To tackle merged detection, we revise Local Contrast Method(LCM) and propose a multi-appearance variant, which exploits tree-like topological information and realizes one threshold for one object. Meanwhile, one-to-many constraint is employed via the proposed extended 0-1 programming, which enables hypothesis selection to handle track exclusions caused by merged detections. Secondly, to alleviate the high complexity caused by dense objects, we consider batch optimization and more rigorous and precise pruning technologies. Specifically, we propose autocorrelation based motion score test and two-stage hypotheses pruning. Experimental results are presented to verify the strength of our methods, which indicates speed and performance advantages of our tracker.

## Introduction

In video content analysis, whether for interpretation, indexing or coding, multi-object tracking performs a prominent role. Recently, dim small object tracking attracts significant interest mainly because of its wide applications, such as military infrared guidance, physical particles analysis and micro-animal observation. The objective of this paper is to develop an algorithm that can detect and track small object in the complex scenario. This algorithm should be capable of accurately locating dim small object, starting and maintaining path and terminating it.

Small object tracking is a complex yet challenging problem. Variable intensity of object, structured backgrounds, electronic noise and frequent occlusions are some obstacles impacting the detection of small object. The unreliability of object detection results in corrupted object measurements and significantly degenerates the performance of the tracker. Besides, ambiguities in the association of consecutive detections to a track can arise due to temporary misdetection, occlusions, crossings and featureless appearance of small object.

### Related works of multi-object tracking methods

Generally, the core problem of multi-object tracking is believed to be the data association, which builds the link between detection and track. From this aspect, most contemporary methods for multi-object tracking belong to two main categories, i.e. one-to-one and many-to-many association. (Since we refer to some traditional studies of multi-target tracking, where measurement means detected objects, we use measurements or detection to mean the same thing).

One-to-one association(or unique neighbor association) means that each measurement could associate with up to only one track. Although many tracking methods belong to this one, we are particularly interested in methods with delay decision, because our application focuses more on tracking performance instead of online result. Such methods use future and previous information together for more reliable association. Multi-Hypothesis Tracking(MHT) [1–4], Multi-Dimensional Assignment(MDA) [5], Semi-Greedy Track Selection(SGTS) [6, 7] are some typical methods using delay decision. Reid proposed the original MHT algorithm [8], which then develops into two categories, namely track-oriented MHT(TOMHT) and hypothesis-oriented MHT(HOMHT). TOMHT means that each hypothesis represents an object track. Many researchers improve MHT by employing efficient assignment methods, such as A* search [4] and Murty’s [9]. MDA [5] expands the bipartite graph matching in MHT to multiply dimensional assignment. SGTS employs a semi-greedy algorithm to get the approximately optimal solution of association assumption. Cox *et al*. present an efficient implementation of MHT using an algorithm due to Murly’s(1968) algorithm [9]. Ren *et al*. propose an efficient GRASP-MHT [10], which integrates a greedy randomized adaptive search procedure (GRASP) within a TOMHT framework.

The second category is many-to-many association or all-neighbor association, where multiple measurements are used for the update of one track. The principal assumption is that each measurement within the threshold gate should contribute to the update of the track with different weights. This method naturally avoids the fixed association as well as exponentially increasing combinations. The typical approaches include Probabilistic Data Association(PDA) and Joint PDA(JPDA) [11]. PDA calculates the association probabilities of the target being tracked for each validated measurement at the current time. This probabilistic or Bayesian information is used in PDAF to account for the measurement origin uncertainty. Besides, JPDA could only track objects with fixed and informed number.

### Motivation

Results from some studies [12, 13] show that the precision of JPDA may be inferior to MHT when handling an excessive amount of objects. Inspired by the idea of TOMHT, we propose a novel tracking approach in particular for dense small object tracking. To achieve accurate and efficient tracking, we must solve the high complexity and merged detection problems brought by dense small object.

#### Merged detection

The average distance between objects becomes shorter for the sake of dense distribution. In that case, occlusion and merged detections would happen more frequently. Tracking algorithms that ignore such a phenomenon can lead to poor performance in multi-object tracking.

Prior work on dense small object tracking with merged detections is sparse, but there are some other works facing the similar problem. (1)One is about unresolved measurement [14, 15], which basically is a resolution problem rather than our segmentation problem. They need to model resolution capability of the used sensor while we don’t. Actually it’s very difficult to get the reliable description of the resolution capability of a given sensor. [14, 15] consider the problem with only two objects, there is no guarantee of such conditions in our application. (2)Severe occlusion produces merged measurement in visual tracking [16, 17], where detection results of pedestrians are bounding boxes. Visual tracking usually handles fewer objects while small object case is denser. Besides, rich appearance information is a very important cue for visual tracking to distinguish objects, but small object is featureless. So, it’s improper to directly employ the above-mentioned approaches for our case.

To tackle the merged detection, we do the followings: (1)For detection, we propose multi-appearance local contrast segmentation(MALC) to detect and clearly segment merged objects. MALC employs the multi-threshold idea and achieves one threshold for one object. (2)Most MHT approaches mainly utilize one-to-one association and neglect merged detection. To deal with merged detections during tracking, we employ one-to-many association to count for the occlusion caused by merged detection, namely that one detection can associate with multiple tracks. Extended 0-1 programming is proposed to achieve our one-to-many association.

#### High complexity

In fact, high complexity is an inherent problem of MHT, especially when dealing with a large number of objects. Dense small object presents exactly such situation. Cluttered background and occlusions cause large ambiguity of detection. Moreover, to tackle some small objects that would change speed rapidly, it is necessary to apply a loose gating threshold. These facts significantly boost the number of hypotheses in MHT.

Although some efficient approaches had been proposed [9, 10], they still can’t achieve a good tradeoff between performance and efficiency for the special scene with dense small objects.

We approach the problem of complexity from two aspects: (1)batch optimization: we find that in MHT a considerable part of the computation is spent on compatibility processing, which accounts for the compatibility between hypotheses and is performed at every time step. In fact, such frequent processing may not be totally necessary, because the compatibility relations won’t change for every time step. Instead, it remains similar for a short period. Appropriately extending the time interval between two consecutive compatibility processes could be beneficial to reduce the computation. (2)hypothesis pruning: batch optimization will increase the hypothesis number, so we design and impose two-stage pruning technologies to control the growth of hypothesis. Meanwhile, to improve the quality of hypothesis, we also propose a new motion score to evaluate the likelihood of hypothesis, which helps for small object tracking.

Batch optimization and new pruning technologies complete each other. Hence, the frequency of compatibility processing is reduced while the scale of hypothesis is still under control.

### Outline and contribution of the paper

The remainder of this paper is organized as follows. In Sec. 2, we present our detection and tracking method for small object. In Sec. 3, the experiments about detection, tracking and verifying new constraint are introduced. Finally, we conclude our paper in Sec. 4.

We make the following contributions: (1)We employ an one-to-many association based constraint for dense small object tracking, and implement it by the proposed extended 0-1 programming; (2)We introduce a two-stage pruning technology and a short-term motion score for hypothesis generation and management, which help to reduce the hypothesis quantity and improve its quality. (3) MALC is proposed for small object detection to handle merged detection. It utilizes a topological tree structure to count for the relationship among local thresholds for different objects. (4)Owing to the efforts of pruning hypotheses and reducing complexity, the implementation of our tracker has an advantage in speed.

## Methods

### Multi-appearance local contrast based detection

We employ the popular tracking-by-detection paradigm [18–23], where objects are first detected by a detector in each frame and then associated across video frames. Thus, detection of small object is our first task. It’s always difficult to clearly distinguish small object from the clutter in background. Moreover, merged object is a challenging problem especially in the scene of dense small object. In this part, we present MALC to tackle merged detections.

As one of the popular methods in dim small object segmentation, local contrast method(LCM) [24] has the great capability for detecting dim object. Besides, its defect is expanding the object using maxpool operation, which could degenerate the performance of tracking for producing more merged detections. In fact, most detectors usually do not take into account the dense object with lots of occlusions. To distinguish touching objects during occlusions, we revise LCM and propose a multi-appearance approach on top of it. We build topological tree structures to organize the objects under different thresholds and the relationship between them.

#### Intention

**Fig 1:**
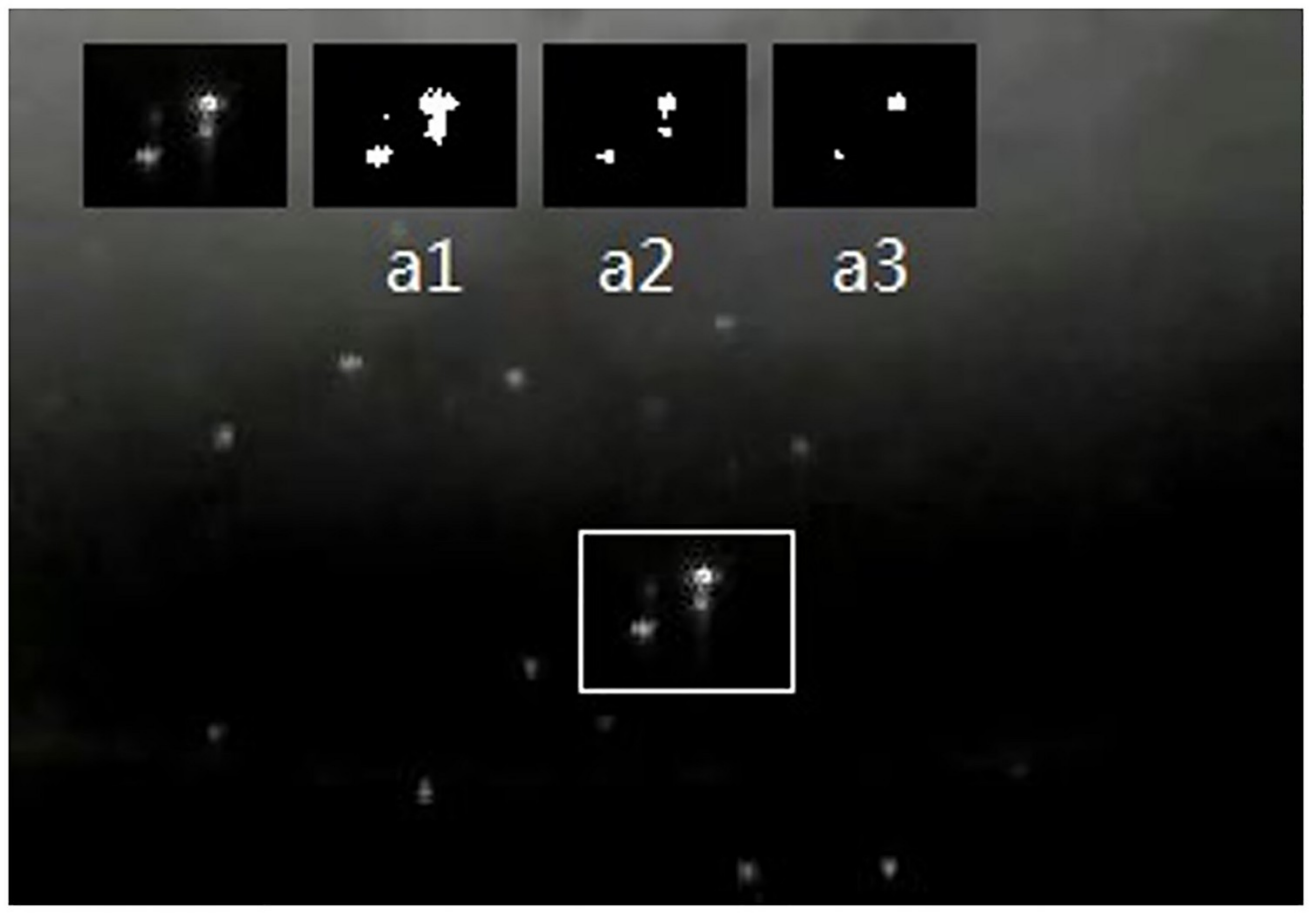
An instance of multiple segmentation slides. In a1, some objects are merged into a single object. As for a3, the segmentation with high threshold loses the sight of certain object.

We provide a simple example in the Fig 1, which indicates that different thresholds for segmentation produce totally different results. As the Fig 1 showing, the low threshold can not distinguish objects and noisy points accurately, which produces merged detection. High threshold would lose some objects. The appropriate threshold should change for the different objects in image.

**Fig 2:**
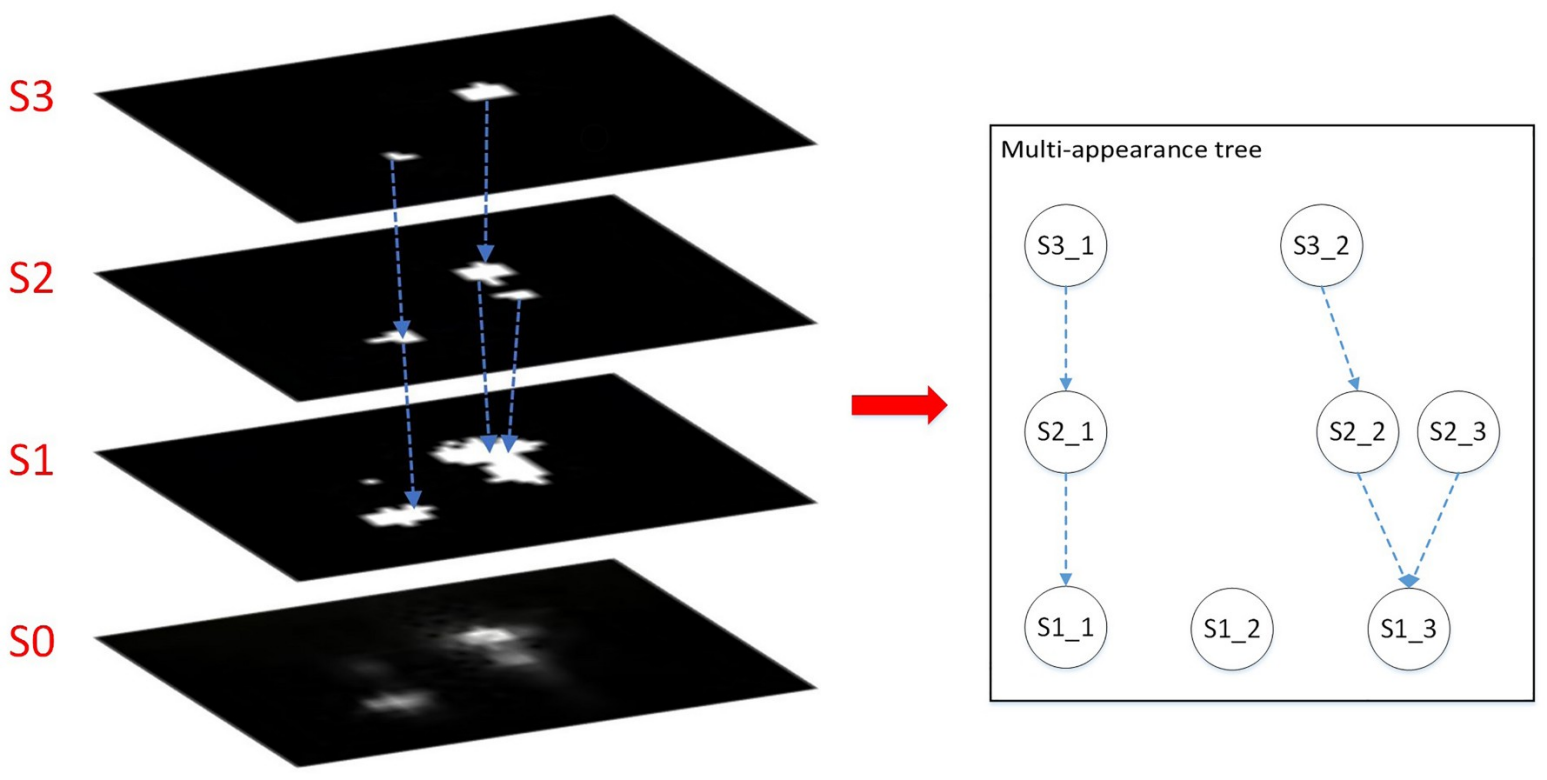
Multi-appearance structure with tree relation.

Each segmentation of the object demonstrates one of its appearances, which contains the contour information under current segmentation and can be interpreted as one slice measurement. From one slice measurement, we can obtain the corresponding object score according to its appearance. Then, we will use those appearance scores to choose the appropriate segmentation. Sequential layers of appearance and affiliated connections together form a multi-appearance tree. Using the example in Fig 2, we explain the affiliated relationship between the layers. As shown in Fig 2, the multi-appearance tree is built to count for the exclusion relation between selections of thresholds for different objects. For instance, the candidate combination for threshold selections in the tree with root node *S*1_3 can only be one from {*S*1_3}, {*S*2_2, *S*2_3} and {*S*3_2, *S*2_3}. That is based on the constraints indicated by the tree structure that one can’t select the *S*1_3 and *S*3_2 simultaneously, since the threshold for a local region is unique. The critical problem in segmentation is to select the most appropriate threshold for each object from all the layers. We introduce Eq 3 as the criteria for segmentation. The goal is to minimize ∑*S*_*appearance*_, i.e. sum of *S*_*appearance*_ for all segmented objects.

#### MALC method

MALC is composed of three stages: For the first stage, the gray image is transformed into the corresponding map using Eqs 2 and 1. In the second stage, a multi-level binarization is performed on the transformed image and the tree structure is built based on the multiple binary images. Finally, during the third stage, we introduce a deep first segmentation selection(Alg. 1), which is executed for each multi-appearance tree.

For the first stage, we use the same Eqs 2 and 1 as C.L.P.Chen’s work [24]:

$$\begin{array}{c} MI_{n}=\frac{1}{N_{u}}\sum_{j=1}^{N_{u}}I_{j} \end{array}$$ (1)

$$\begin{array}{c} C_{n}=\frac{I_{n}^{2}}{MI_{n}} \end{array}$$ (2)

where *MI*_*n*_ is the mean intensity of the neighbor area of *I*_*n*_. The neighbor area of one pixel is the region between outer square windows and inner windows with current pixel at center. The outer and inner width are set to 9 and 3, respectively. *N*_*u*_ is the number of pixels in the neighbor area, and *I*_*j*_ is the gray level of the *j*th pixel in neighbor area. *I*_*n*_ represents the gray level of the central pixel and *C*_*n*_ is the final correspond value. Unlike original approach, we remove the minimum search and keep the sharp edge of object.

Secondly, we calculate the mean and standard deviation of *C*_*n*_. The global threshold is the sum of mean and *K* times of standard deviation. Using global threshold as the reference middle value, *n* layers’ thresholds are set with equal interval (*n* and the interval are pre-defined parameters). For each threshold, the corresponding binarization is performed. The tree with the relationship between objects from adjacent layers is built.

**Algorithm 1 Deep first segmentation selection**

**Require**: *n*_*root*_

**Ensure**: *S*_*root*_

1: *S*_*child*_*score*_*sum*_ = 0

2: Calculate *S*_*appearance*_ according to Eq 3.

3: **for** each *n*_*child*_ satisfying that *n*_*child*_ is child node of *n*_*root*_ **do**

4:  *S*_*child*_*score*_*sum*_ ← *S*_*child*_*score*_*sum*_ + Deep first branch adjustment(*n*_*child*_)

5: **end for**

6: **if** *S*_*child*_*score*_*sum*_ < *S*_*appearance*_ **then**

7:  *S*_*root*_ ← *S*_*child*_*score*_*sum*_

8: **else**

9:  mark *n*_*root*_ as candidate node

10:  *S*_*root*_ ← *S*_*appearance*_

11: **end if**

For the third stage, we introduce Eq 3, which provides each node in the tree with an appearance score, to evaluate a segmented object. We define the appearance score as the product of three score components, namely intensity, shape and bubble punishment terms:

$$\begin{array}{cc} & S_{appearance}=S_{intensity}S_{shape}S_{bubble\_punishment} \end{array}$$ (3)

$$\begin{array}{cc} & S_{intensity}=\sqrt{\frac{\sum_{x,y\in O}{\left(I_{x,y}-\bar{I}\right)}^{2}}{n}} \end{array}$$ (4)

where *S*_*intensity*_ evaluates the object by the consideration that standard deviation of intensity will maintain higher stability inside the separated object.

$$\begin{array}{cc} & S_{shape}=\frac{\sum_{x,y\in O_{edge}}^{}{\left(d_{x,y}-\bar{d}\right)}^{2}}{n} \end{array}$$ (5)

$$\begin{array}{cc} & \bar{d}=\frac{1}{n}\sum_{x,y\in O_{edge}}d_{x,y} \end{array}$$ (6)

$$\begin{array}{cc} & d_{x,y}=\sqrt{{\left(x-\bar{x}\right)}^{2}+{\left(y-\bar{y}\right)}^{2}} \end{array}$$ (7)

We use *S*_*shape*_ to measure the impact of object geometric shape. Accurate segmentation should impel the object contour to be smooth and regular. *S*_*shape*_ is defined as the variance of distance between edge pixel and object center. *O*_*edge*_ is the set of edge pixels, and *n* is the number of edge pixel. *d*_*x*, *y*_ is the distance between a certain pixel in object and the object center($\bar{x},\bar{y}$), $\bar{d}$ is the mean of *d*_*x*, *y*_. And a punishment factor *S*_*bubble*_*punishment*_ is used for the regularization:

$$\begin{array}{c} S_{bubble\_punishment}=N_{bubble}+1 \end{array}$$ (8)

where bubble, the non-detected pixels in the object, indicates inappropriate segmentation.

The goal is to minimize ∑*S*_*appearance*_, i.e. sum of *S*_*appearance*_ for all segmented objects. We propose Alg. 1 to perform traverse and search for the optimal segmentation with minimum ∑*S*_*appearance*_. Alg. 1 compares the score of current node with the score sum of children to choose the proper segmentation (current level or deeper child level). Then, those chosen nodes are marked. Finally, only those top marked nodes, with no other marked nodes in the paths between them and root, are used to compose the optimal segmentation. We use a breadth-first search algorithm to pick out the top marked nodes.

### Multi-hypothesis tracker

#### Tracking framework

**Fig 3:**
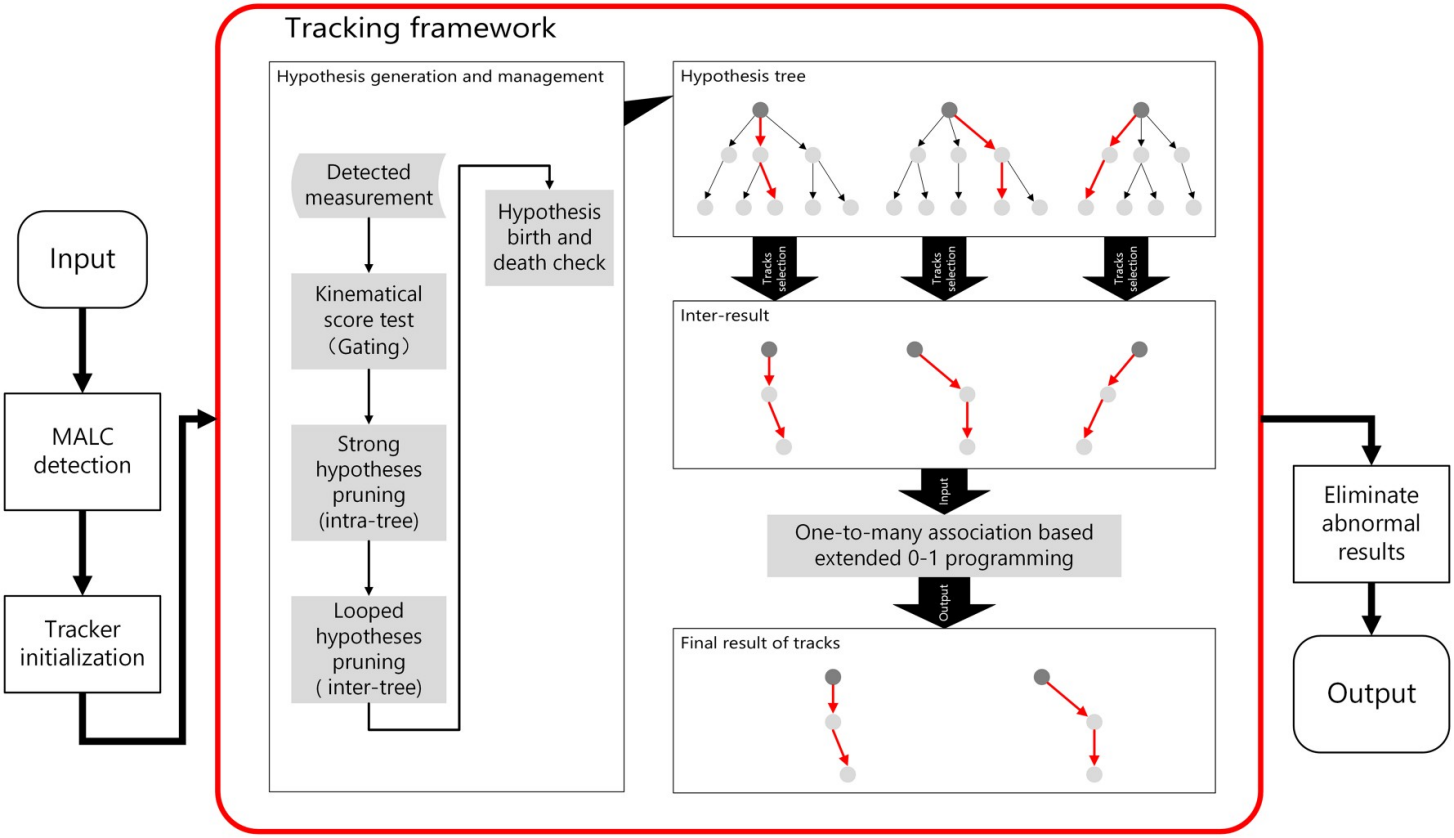
The proposed tracking framework.

The tracking framework is illustrated as the working flow diagram in Fig 3 where hypothesis generation and selection are performed. Hypothesis generator produces the candidate hypotheses of every tree, which are sent to the hypothesis selection component as input data. Then, extended 0-1 programming would determine the set of preserved hypotheses according to the compatible relationship among hypotheses. Extended 0-1 programming is a ‘soft’ method with more flexibility to handle complex occlusion circumstances. In other words, it relaxes the constraint when treating some incompatible tracks.

#### Hypothesis generation and management

We integrate some technologies for hypothesis generation and management. Since our tracking method is designed to be of batch optimization, 0-1 programming will not be executed at every frame but over a larger span. Thus, the hypothesis tree will grow to an uncontrollable scale if no special restriction measure is employed. The main function of the proposed technologies in this part is to decrease the number of selected hypotheses while preserving reliable ones.

In this section, we first present the mathematic expression form for tracking description. Then, along with the pipeline of hypothesis generation and management in Fig 3, we introduce our new score definition, gating and pruning technologies.

Tracking description. Assuming a sensor scans the surveillance region periodically, the set of detections received at frame *t* is denoted by *D*(*t*):

$$\begin{array}{c} D\left(t\right)={\left\{d_{i}^{t}\right\}}_{N_{t}}^{i=0},t=1,2,...,N \end{array}$$ (9)

where *N* is the number of frames, ${\left\{d_{i}^{t}\right\}}_{N_{t}}^{i=0}$ is the *i*th detection received at frame *t*, and *N*_*t*_ is the number of detections received at frame *t*. In addition, a dummy observation $d_{0}^{t}$ is defined for each frame *t* to denote possible missed detections. A track hypothesis (we would use hypothesis for short in following content) $T_{j}^{t}$ at frame *t* is defined as a sequence of observations:

$$\begin{array}{cc} & T_{j}^{t}=\left(d_{j_{1}}^{1},d_{j_{2}}^{2},...,d_{j_{t}}^{t}\right),d_{j_{n}}^{n}\in D\left(n\right) \end{array}$$ (10)

$$\begin{array}{cc} & O_{l}^{t}=\left(T_{1}^{t},T_{2}^{t},...,T_{n_{l}}^{t}\right) \end{array}$$ (11)

$$\begin{array}{cc} & O^{t}=\left(O_{1}^{t},O_{2}^{t},...,O_{n}^{t}\right) \end{array}$$ (12)

This definition constitutes a restriction that one track can contain at most one detection at a particular frame. $O_{l}^{t}$ is the set of hypothesis tracks with all the $T_{n}^{t}$ in it possessing same root(in one connected tree). *O*^*t*^ is the set of $O_{l}^{t}$, which represents all tree hypotheses at frame *t*.

Score based on autoregressive model of acceleration. Firstly, we defined *S*_*AA*_ to represent an autoregressive model of acceleration, which would be used for score test gating.

$$\begin{array}{cc} & S_{AA}\left(T_{j}^{t}\right)=\frac{S_{AA}\left(T_{j}^{t-1}\right)\left(N_{C}^{j}-1\right)+MD_{d_{i}^{t},P_{j_{t-1}}^{t}}}{N_{C}^{j}+1} \end{array}$$ (13)

$$\begin{array}{cc} & MD_{d_{i}^{t},P_{j_{t-1}}^{t}}=mahaldist\left(V_{d_{i}^{t}},V_{T_{j}^{t-1}}\right) \end{array}$$ (14)

where the following notations are used:

$N_{C}^{j}:$ depth of hypothesis *j*, which will not exceed the depth of practical hypothesis tree.

$S_{AA}\left(T_{j}^{t}\right):$ score of leaf hypothesis *j* at frame *t* for autoregressive model of acceleration;

$V_{d_{i}^{t}}:$ the velocity of $d_{i}^{t}$ at frame *t* assumed association between $d_{i}^{t}$ and $T_{j}^{t-1}$ is built;

$d_{i}^{t}:$ detection *i* at frame *t*;

$P_{j_{t-1}}^{t}:$ prediction deduced from the $T_{j}^{t-1}$;

*mahaldist*(⋅): function to get mahalanobis distance.

The velocity and prediction mentioned above are acquired through correction of Kalman filter. After transforming $S_{AA}\left(T_{j}^{t}\right)$, we can get following autoregressive formula of $MD_{d_{i}^{l},P_{j_{l-1}}^{l}}$, where *l* is the frame number. The initial $S_{AA}\left(T_{j}^{t}\right)$ is set as zero, so the constant term of this autoregressive formula is zero.

$$\begin{array}{cc} & S_{AA}\left(T_{j}^{t}\right)=\sum_{l=1}^{t}a_{l}MD_{d_{i}^{l},P_{j_{l-1}}^{l}} \end{array}$$ (15)

$$\begin{array}{cc} & a_{l}=\frac{2}{N_{C}+1}{\left(\frac{N_{C}-1}{N_{C}+1}\right)}^{t-l} \end{array}$$ (16)

The coefficient *a*_*l*_ decays along with the *l*, and motion information from recent time will contribute more.

Score test for association gating. For each leaf node in hypothesis tree, a $S_{AA}\left(T_{j}^{t}\right)$ is calculated and maintained for every time step. Then, we use the Eq 18 to filter out(or called gating) unsatisfying hypotheses before branch growth. *A*_*ij*_ = 0 means the corresponding association won’t pass gating.

$$\begin{array}{cc} & S_{ST}=\left|MD_{m,P}-S_{AA}\left(T_{j}\right)\right| \end{array}$$ (17)

$$\begin{array}{cc} & A_{ij}=\{\begin{array}{cc} 1 & S_{ST}<th_{n},\\ 0 & \text{else}. \end{array} \end{array}$$ (18)

$$\begin{array}{cc} & th_{n}=\{\begin{array}{cc} (\alpha -N_{s})\beta & (\alpha -N_{s})>\gamma ,\\ \delta & \text{else}. \end{array} \end{array}$$ (19)

*th*_*n*_: multi-stage threshold for score test;

*A*_*ij*_: association filter mask between detection *i* and hypothesis *j*, *A*_*ij*_ = 1 indicates permission of association between detection *i* and hypothesis *j*;

*N*_*S*_: the number of sustaining frames;

*α*, *β*, *γ*, *δ*: parameters for multi-stage threshold.

**Fig 4:**
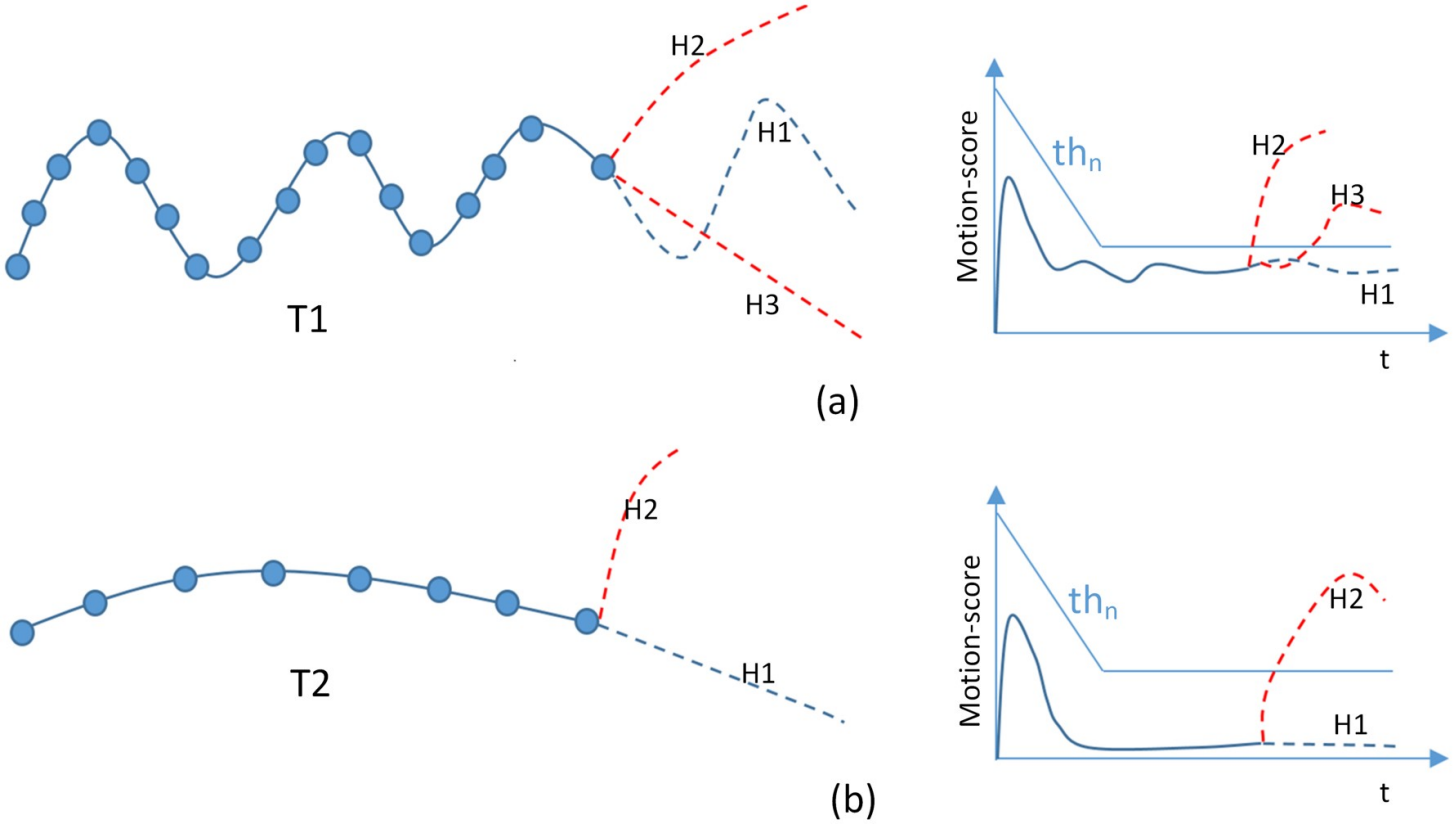
Illustration of score testing. Motion score used here is *S*_*ST*_. The fold line above the score curve is our multi-stage threshold *th*_*n*_.

We define *S*_*ST*_ to describe the strength for a track to maintain its previous motion pattern, which is based on the assumption that absolute acceleration of a track will change very slowly in a certain degree. *MD*_*m*, *P*_ reflects the difference between prediction velocity and practical one, namely absolute acceleration. As Eq 15 shows, $S_{AA}\left(T_{j}^{t}\right)$ is the previous weighted acceleration. |*MD*_*m*, *P*_ − *S*_*AA*_(*T*_*j*_)| is the absolute difference between previous weighted absolute acceleration and current one, which reflects the deviation between current movement and expected one. Eq 18 would reject a hypothesis with sudden and big acceleration change. Therefore, our score testing is capable to capture the coarse pattern of movement. Multi-stage threshold *th*_*n*_ is controlled by parameters *α*, *β*, *γ* and *δ*, which is a polyline as in Fig 4. The loose threshold at beginning facilitates the start of new object because new object may be unstable and come with high *S*_*ST*_. When object trajectory becomes stable, following small threshold helps to retain robust objects and discard unreliable ones.

We use Fig 4 to illustrate score test. Threshold *th*_*n*_ is the polyline with two stages. Once the *S*_*ST*_ crosses the threshold line, the corresponding hypothesis will be abandoned. For the motion score of *T*1 (the curve in Fig 4(a) with frequently changing motion pattern), three potential hypotheses are given. The diagram of the corresponding motion score is drawn in the right side. *H*1 and *H*2 are rejected since these two smooth tracks deviate far away for its original winding motion pattern. In Fig 4(b), *H*2 is distinguished from *T*2 by its sudden change of movement while *H*1 follows previous smoothness.

Hypothesis score. To indicate track’s likelihood, each hypothesis is assigned with a hypothesis score, which is used for hypothesis selection and pruning. The score of hypothesis *j* at frame *t* is defined as follows:

$$\begin{array}{c} S\left(T_{j}^{t}\right)=\omega_{LTM}S_{LTM}\left(T_{j}^{t}\right)+\omega_{STM}S_{STM}\left(T_{j}^{t}\right) \end{array}$$ (20)

where $S_{LTM}\left(T_{j}^{t}\right)$ and $S_{STM}\left(T_{j}^{t}\right)$ are the scores of hypothesis *j* in the consideration of long-term motion and short-term motion respectively. *ω*_*LTM*_ and *ω*_*STM*_ are the weights for long-term motion and short-term motion.

$S_{LTM}\left(T_{j}^{t}\right)$ is the original score formulation [8] in traditional MHT. We introduce $S_{STM}\left(T_{j}^{t}\right)$ to capture the short-term motion and to enhance the sensitivity of $S(T_{j}^{t})$ for rapid motion variation. The original score is a slowly changing value and will increase with time for the potential track. After a long period of update, it reaches a rather high level and partly loses its sensitivity for motion change. Although some methods like SQRT employ a threshold to detect the change, this hard threshold measure can’t provide enough agile information to reflect short motion. $S_{LTM}\left(T_{j}^{t}\right)$ indicates the likelihood of hypothesis from a long-term and overarching perspective.

Firstly, we revise $S_{LTM}\left(T_{j}^{t}\right)$, which uses cumulative log-likelihood ratio.

$$\begin{array}{c} S_{LTM}^{t}=\sum \Delta S_{LTM}^{i}\left(T_{j}^{t}\right) \end{array}$$ (21)

If hypothesis $T_{j}^{t-1}$ is associated with detection $d_{i}^{t}$ at frame *t*, then the increment of hypothesis score is given by:

$$\begin{array}{c} \Delta S_{LTM}^{i}\left(T_{j}^{t}\right)=\{\begin{array}{cc} \log(\frac{p\left(d_{i}^{t}|T_{j}^{t-1}\right)P_{D}}{\lambda_{fa}+\lambda_{nt}}) & i\neq 0,\\ \log(1-P_{D}) & i=0. \end{array} \end{array}$$ (22)

where the following notations are used:

$d_{i}^{t}:$ vector of detection *i* at frame *t*;

*p*(⋅): the probability density function (PDF) of detection $d_{i}^{t}$ conditioned on the one-step prediction of hypothesis $T_{j}^{t-1}$;

*P*_*D*_: detection probability;

λ_*fa*_: the expected number of false alarms per unit volume of the detection space per frame(spatial density of clutter);

λ_*nt*_: spatial density of new targets.

The initial hypothesis score $\Delta S_{LTM}^{i}\left(T_{j}^{t}\right)$ is given as $\log(\frac{\lambda_{nt}}{\lambda_{fa}})$.

Then, we define $S_{STM}\left(T_{j}^{t}\right)$ as Eq 23.

$$\begin{array}{cc} & S_{STM}^{i}\left(T_{j}^{t}\right)=\sum_{n=1}^{IC}\log\left(\frac{th_{n}-S_{ST}}{S_{ST}}\right) \end{array}$$ (23)

We use the ratio of *S*_*ST*_ to represent the short-term score. With *S*_*ST*_, we design a short-term score according to the variability of recent acceleration, which assumes a constant acceleration model. Since we abandon those hypotheses with *S*_*ST*_ higher than *th*_*n*_ in score test, so *th*_*n*_ would be the upper limit of *S*_*ST*_. $\log(\frac{th_{n}-S_{ST}}{S_{ST}})$ can be roughly regarded as a likelihood rate(LLR) of *S*_*ST*_ toward acceleration smoothness. Then *S*_*STM*_ is the accumulation of this LLR, which reflects recent acceleration deviation trend. *IC* is the number of effective emergencies, which counts the times when a valid detection is assigned to the current object.

Hypothesis pruning. After score test, two stages of pruning are carried out to remove incompatible hypothesis.

The first stage is strong hypotheses pruning, which is performed between different trees. A comparison between two hypotheses, one with the highest score and the other one with the highest *IC*, is conducted. They are the historic best one and the temporary best one respectively. Hypotheses would be incompatible if they share too many detections in the recent period. We apply such operation because those wrong strong hypotheses could become more stubborn if not being handled at the beginning.

For the second stage, we use Alg. 3 to detect loop path and solve it by pruning low-probability hypothesis and retaining only one. Loop path means that two hypotheses start from same node and end with same node, but have different paths. This phenomenon may bring the exponential growth of hypothesis number if no special treatment is employed. Immediate pruning is performed in our algorithm so that unnecessary ambiguity is removed before development.

**Algorithm 2 Pruning stage 1**: **Strong hypotheses pruning(inter-tree)**

**Require**: *O*^*t*^

**Ensure**: *O*^*t*^

1: **for** each $d_{n}^{t}\in D\left(t\right)$ **do**

2:  **for** each $T_{i}^{t}$ satisfying $d_{n}^{t}\in T_{i}^{t}$ **do**

3:   find $T_{sm}^{t}$ with max $S(T_{ms}^{t})$.

4:   find $T_{mic}^{t}$ with max $IC(T_{mic}^{t})$.

5:   **if** $T_{mic}^{t}\neq T_{sm}^{t}$ **and** the number of shared detections exceeds tree depth **then**

6:    **if** $S\left(T_{mic}^{t}\right)>=S\left(T_{ms}^{t}\right)$ **then**

7:     $O_{i}^{t}\leftarrow O_{i}^{t}-\left\{T_{ms}^{t}\right\}$

8:    **else**

9:     $O_{j}^{t}\leftarrow O_{j}^{t}-\left\{T_{mic}^{t}\right\}$

10:    **end if**

11:   **end if**

12:  **end for**

13: **end for**

**Algorithm 3 Pruning stage 2**: **Loop hypotheses pruning(intra-tree)**

**Require**: *O*^*t*^

**Ensure**: *O*^*t*^

1: **for** each $O_{l}^{t}\in O^{t}$ **do**

2:  **for** each $T_{i}^{t}\in O_{l}^{t},T_{j}^{t}\in O_{l}^{t}$ **and** $T_{i}^{t}\neq T_{j}^{t}$ **do**

3:   **if** $d_{n}^{t}\in T_{i}^{t}$ **and** $d_{n}^{t}\in T_{j}^{t}$ **then**

4:    **if** $S_{AA}\left(T_{i}^{t}\right)>=S_{AA}\left(T_{j}^{t}\right)$ **then**

5:     $O_{l}^{t}\leftarrow O_{l}^{t}-\left\{T_{j}^{t}\right\}$

6:    **else**

7:     $O_{l}^{t}\leftarrow O_{l}^{t}-\left\{T_{i}^{t}\right\}$

8:    **end if**

9:   **end if**

10:  **end for**

11: **end for**

For inter-tree hypothesis pruning, hypothesis score is employed as the criteria. As for intra-tree pruning, *S*_*AA*_ is used, which is the proposed autoregressive model of acceleration.

Birth and death of hypothesis. Those detections that don’t link to any hypothesis would be regarded as the starts of new objects. We employ SQRT test [8] to terminate a hypothesis that has no linked detection for the update. We rank the terminated hypotheses at the last frame of a batch by their $S(T_{j}^{t})$(Eq 20). Then we preserve only top 20 percent for hypothesis selection to reduce computation.

#### Hypothesis selection as an extended 0-1 programming

**Fig 5:**
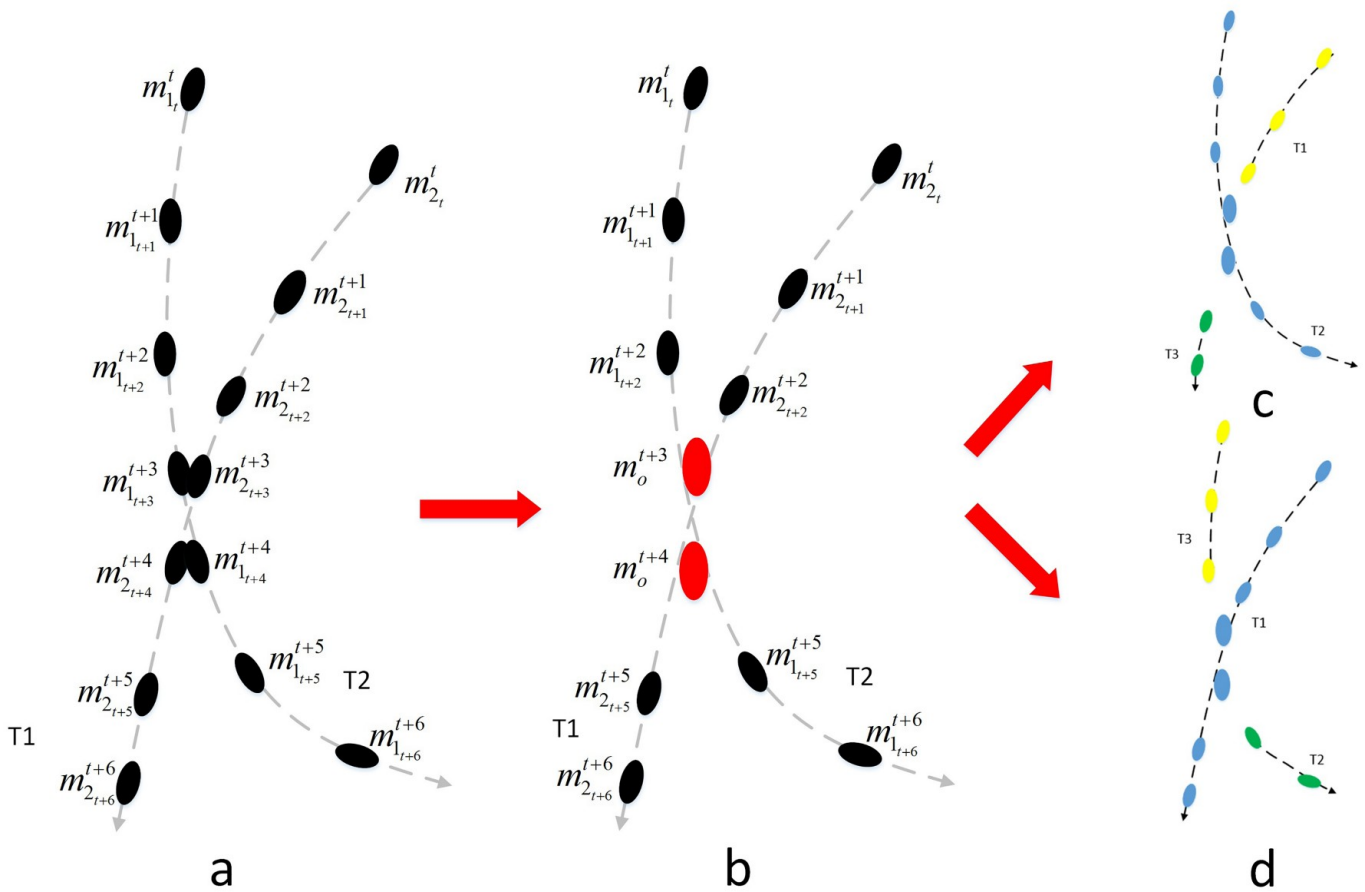
Two tracks with merged detections.

When handling the merged detections in dense small object tracking, one-to-many association may be more appropriate than one-to-one. Most of past studies assume that at most one object is associated with each detection. As for the occlusion with approximative motion, two or more objects are detected as only one detection. As Fig 5 illustrates, if we insist the assumption of one-to-one association, big gaps may occur since some tracks lose necessary detections for association, which would influence the correct formation of tracks. In comparison, one-to-many association facilitates the complete formation of each hypothesis through the occlusion.

Formula for one-to-many association. Track selection is naturally a binary linear programming problem in the view of using binary variable to control the selection of hypothesis. The traditional formula of one-to-one association (such as in [25]) is like following:

$$\begin{array}{c} \min C=\min\sum_{n}\xi_{T_{n}}C_{T_{n}} \end{array}$$ (24)

$$\begin{array}{c} s.t.\;\xi_{T_{i}}+\xi_{T_{j}}<=1,\;T_{i}\cap T_{j}\neq \emptyset \;and\;i\neq j \end{array}$$ (25)

$$\begin{array}{c} \xi_{T_{n}}\in \left\{0,1\right\} \end{array}$$ (26)

where $C_{T_{n}}$ is defined as the cost of hypothesis *n*. $\xi_{T_{i}}$ is a binary representation of *T*_*i*_. To efficiently apply one-to-many association, we redesign an extended 0-1 linear programming with adjunctive binary variable *I*_*ij*_ as following formula:

$$\begin{array}{c} \min C=\min\sum_{n}\xi_{T_{n}}C_{T_{n}}+\sum_{i,j}I_{ij}C_{I_{ij}} \end{array}$$ (27)

$$\begin{array}{c} s.t.\;I_{ij}\geq \xi_{T_{i}}+\xi_{T_{j}}-1 \end{array}$$ (28)

$$\begin{array}{c} I_{ij}\leq \left(\xi_{T_{i}}+\xi_{T_{j}}\right)/2 \end{array}$$ (29)

$$\begin{array}{c} I_{ij},\xi_{T_{n}}\in \left\{0,1\right\} \end{array}$$ (30)

where *I*_*ij*_ represents the incompatibility between hypothesis *i* and *j*. *I*_*ij*_ will equal to 1 while hypothesis *i* and *j* own same detection. *I*_*ij*_ = 0 indicates that hypothesis *i* and *j* are totally irrelevant with each other. The constraints given by Eqs 28 and 29 ensure that while *T*_*i*_ and *T*_*j*_ are both selected, *I*_*ij*_ will be 1, otherwise *I*_*ij*_ equals to 0. $C_{I_{ij}}$ represents the cost of incompatibility to select both hypothesis *i* and *j*.

We utilize adjunctive binary variables *I*_*ij*_ to count for one-to-many assignment. Without the *I*_*ij*_, the problem would turn into a quadratic one. This is because it will be necessary to use quadratic term like $\xi_{T_{i}}\xi_{T_{j}}C_{I_{ij}}$ to count for the flexible compatibility cost. It is much easier to solve a linear programming problem than a quadratic one. Besides, via the introduction of *I*_*ij*_ and $C_{I_{ij}}$, the exclusion restriction between incompatible hypotheses becomes roughly controllable. We slightly loosen exclusion restriction to encourage longer hypothesis formation.

Details of extended 0-1 linear programming. We use following criteria as the cost of hypothesis.

$$\begin{array}{c} C_{T_{n}}=-KS\left(T_{n}^{t}\right) \end{array}$$ (31)

where $S(T_{n}^{t})$ is the score of leaf hypothesis *T*_*n*_ at frame *t*. *K* is used as adjustment coefficient. As for the cost of incompatible hypotheses couple, we use following formula:

$$\begin{array}{c} C_{I_{ij}}=\{\begin{array}{cc} N_{I}^{ij}C_{IN} & N_{I}^{ij}<N_{T},\\ \infty & \text{else}. \end{array} \end{array}$$ (32)

where $N_{I}^{ij}$ is denoted to be the number of incompatible detections between *T*_*i*_ and *T*_*j*_. *C*_*IN*_ is a constant coefficient to count for incompatibility of single detection sharing. *N*_*T*_ is the threshold of tolerable maximum number. If $N_{I}^{ij}$ exceeds *N*_*T*_, a positive infinite value will be assigned to $C_{I_{ij}}$ to indicate that at most one in *T*_*i*_ and *T*_*j*_ can be selected.

Then, after the building of 0-1 programming for each batch of frames, we use lpsolve to solve this problem. Owning to the rational assumptions and optimized constraints, solving progress is not complex. Detailed time consumption and analysis will be presented in the next section.

## Results

In this section, we describe several experiments to verify the performance of our segmentation and tracker. The detailed analysis will be presented to prove the capacity of the proposed method.

### Comparing methods

Firstly, we list comparison methods and its parameter settings. Two parts of the experiment were performed, i.e. segmentation and tracking.

#### Segmentation methods

- **LCM**: Using contrast based template operator would help to extract salient points, and also will be conducive to local optimization [24].
- **Top-hat**: Top-hat operation can extract small elements and details from image, which had been found truly useful for small object detection.
- **MPCM**: Multiscale patch-based contrast measure(MPCM) [26] is also a contrast measure-based method.

#### Tracking methods

- **Cox’s MHT**: We use cox’s implementation [9] of MHT with default parameter settings. We also compared the speed of cox’s with our method when running on the same platform and implementing in the same language (C++).
- **SGTS**: The number of semi-greedy solutions generated before selection was set at 60.
- **MDA**: The duality gap of the termination criterion was set to 0.02. We defined the maximum number of iterations to 100. The Lagrange multiplier updating scheme applied was the heuristic price update, because it’s believed to be more efficient and can fully exploit the structure of the intermediate feasible solutions found by the Auction algorithm.
- **GRASP-MHT**: Greedy randomized adaptive search was applied in multi-object tracking by Murphey *et.al* [27] and Robertson *et.al* [28]. We used a MATLAB implementation from ren’s [10], where GRASP is used as an engine of hypothesis generation in the MWISP formulated TOMHT [10]. Parameters *nv*, *np*, and *nitr*, which were used to control the amount of computation in candidate construction, were set to 20, 20, and 3, respectively.

### Performance metrics

We employ various metrics to evaluate the performance of our detection and tracking method. Some traditional metrics like OSPA-T [29] are used in evaluation. To evaluate the methods by a more comprehensive and representative way, we also utilize some metrics from the field of visual multi-object tracking such as IDSW, MOTA [30]. The most important metrics for tracking include Optimal Sub-pattern Assignment Distance(OSPA-T), Number of Identity Switch(IDSW) and Multiple Object Tracking Accuracy(MOTA). As for detection, Detection Rate(DR) and Standard Deviation of Detection Rate(DR-STD) are some representative indicators. We introduce detailed information of all performance metrics in S1 Appendix.

We use up arrow ↑ to represent that higher score is better result. The opposite of that, down arrow ↓, means preference to lower score.

### Dataset

Two datasets are used in our experiments, denoted as Larva and Verti_Hat respectively.

#### Larva

Three segments of video data with movements of micro-animal are used in this dataset. They were captured under different conditions. The object number in Larva_s2 is much higher than others. In Larva_s3, focal length changes for several times, namely focus drift. Image could suddenly become blurry in few frames, and detection failure happens more frequently.

**Fig 6:**
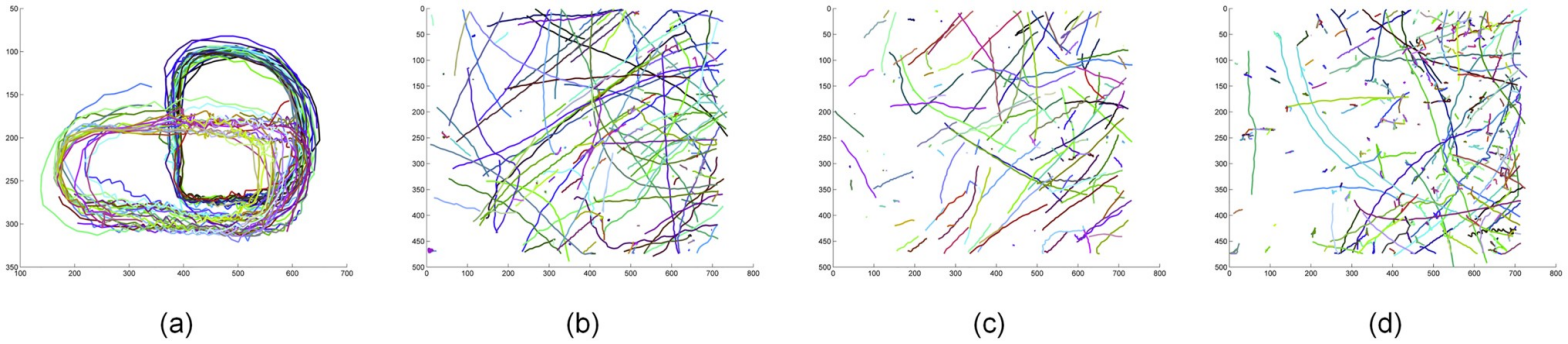
Ground-truth trajectories. (a)Verti_Hat (b)Larva_s1 (c)Larva_s2 (d)Larva_s3.

We sampled the video images at a regular interval for experiments. The ground truth is produced partly based on the tracking results of the few tracking methods mentioned before. The videos used for pre-designation are of full frame without down sampling, so it’d be of less ambiguity because more information is provided to fill the uncertain gap between time steps. Using those results as the reference we checked and corrected the trajectories manually, especially focusing on key frames where occlusions or false alarms happen. The ground-truth trajectories are presented in Fig 6b, 6c and 6d.

#### Verti_Hat

With a vertical view, around 60 persons with hats walk around in this pedestrian video. After adding disturbance and removing some detections randomly, this scene becomes considerably difficult. This video comes from [10]. We intercepted the first 600 frames of the pedestrian video, then down sampled it at 5Hz as the author did. Finally, 120 frames of images were used in this scenario test. Unlike only 60 frames used in [10]’s experiment, we used twice longer(not frequency) sequence to produce more fair evaluation.

### Parameters

The depth of hypothesis tree is set to 6, parameters for multi-stage threshold are set as *α* = 20, *β* = 0.8, *γ* = 10, *δ* = 6. *ω*_*LTM*_ and *ω*_*STM*_ are both 1. As for parameters in *S*_*STM*_, *P*_*D*_ = 0.9, λ_*fa*_ = 1*e*^−6^, λ_*nt*_ = 1*e*^−8^. *K*, *C*_*IN*_, *N*_*T*_ in the programming part are set to 5,1,5 respectively.

The batch length is set to 40 with an overlapped window of 20. For each batch, we only decide the result of front 20 frames while selection procedure can take into account the information from 40 frames. This could help to provide more robust performance for knowing temporally wider information.

Usually, if the distance between a ground truth and a detected position is less than a threshold, then the detection is declared as being correct [31]. We chose 15 as the distance threshold according to proximate increasing of proportion compared with [24]. For some visual tracking metrics assuming that objects occupy certain space, we treat each object as in the box with an radius of 15.

### Segmentation evaluation with tracking metrics

We arrange a detection experiment to test our MALC segmentation compared with other popular methods.

First of all, we used F-score to pre-evaluate three detection methods. F-score is widely used for evaluation with definition of *F* = 2*recall*precision/(*recall* + *precision*). A best K was chosen for each detection method according to the test of F-score on part of Larva dataset. Then, we got best *K* = 1.8, 4.8, 3.5, 5.8 for LCM, MPCM, MALC and Top-hat respectively. In fact, the *K* ∈ [3, 5] recommended in [24] performs poor result in dense object scenario with a small Detection Rate(DR). Dense objects occupy more image space, so a new *K* should be determined via sample test. We used the best *K* for each detection method to ensure that every method yields its best result.

**Fig 7:**
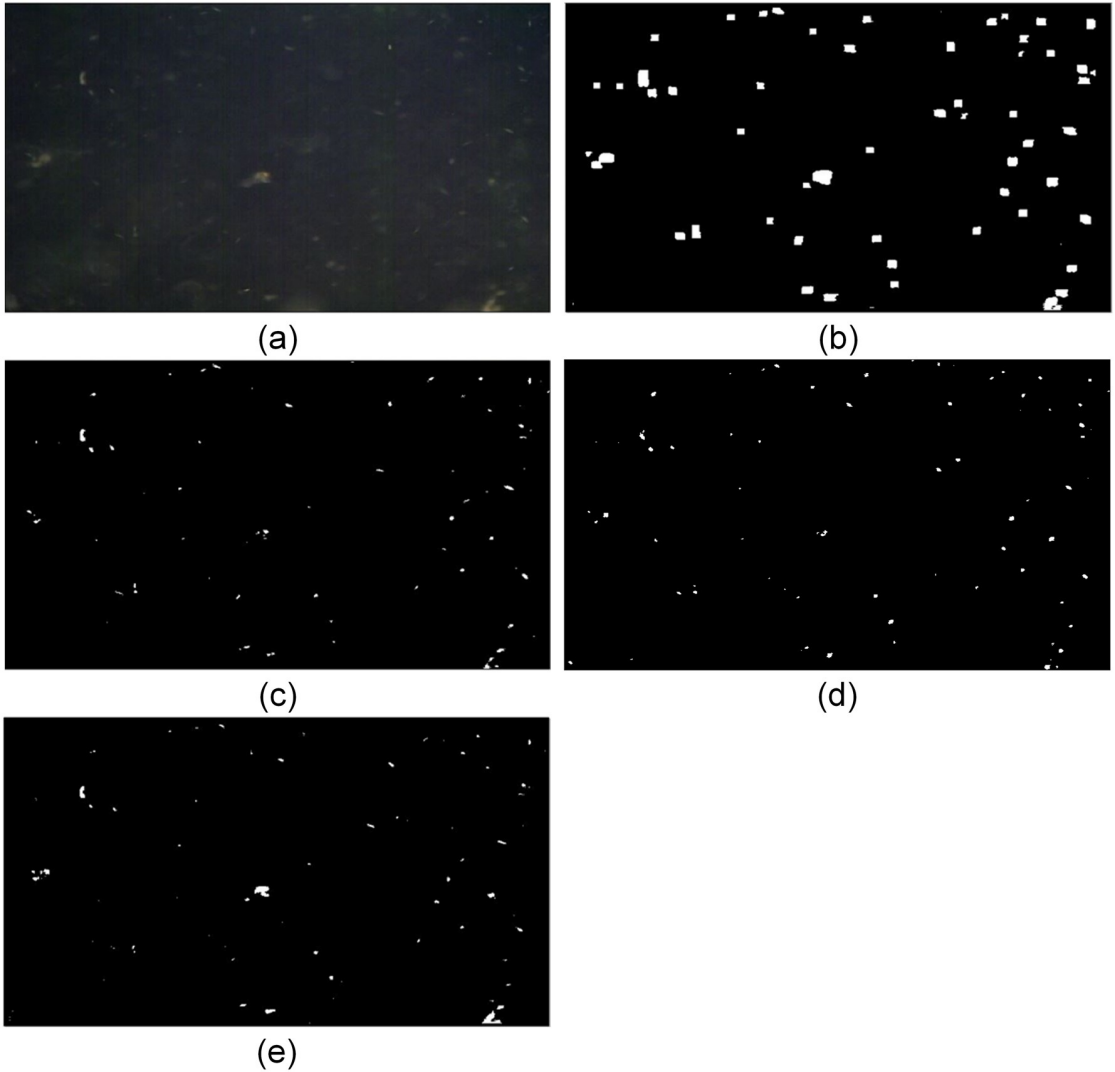
Segmentation results. (a)Original image (b)LCM (c)Top-hat (d)MPCM (e)MALC.

Several metrics are listed in Table 1, which include some tracking metrics to evaluate detection from the view of the whole tracking procedure. Considering the real purpose of this paper, i.e. small object tracking, evaluating the final tracking consequence would be more objective and appropriate by integrating segmentation as part of tracking. The tracking procedure used for three detection methods is totally identical with the same settings. In fact, according to the F-score, our segmentation is not superior to LCM. However, LCM exposes some problems and gets lower MOTA in tracking result. As the response map of LCM in Fig 7 shows, object dilation caused by max-pooling operation produces more merged detection, which could be beneficial to remove false alarms but also degenerates the performance of tracking. That is the reason we develop new segmentation to resist merged detection and improve the results of tracking.

**Table 1:** Performance comparison between our segmentation method and others for the Larva_s1 dataset.

Method	Detection Metrics			Tracking Metrics	
	DR(↑)	DR-STD(↓)	FA(↓)	OSPA-T(↓)	MOTA(↑)
MALC	0.900	0.0473	29.0	13.5	67.3
Top-Hat	0.793	0.0524	24.9	14.6	48.7
MPCM	0.801	0.0690	25.6	15.4	49.6
LCM	0.846	0.0653	13.5	16.8	52.6

Table 1 shows that our method achieves the best DR and lowest DR-STD. Meanwhile, it yields more false alarms. According to the final tracking result, MALC improves MOTA [32] by more than ten percent in comparison with using LCM. For other tracking metrics, MALC gets best scores too. The performances of MPCM and Top-hat is not very good in our scene. The result demonstrates the detection capability of MALC and excellent cooperation between the proposed detection and tracking method.

### Tracking evaluation

We present a series of experiments in this part including tests for one-to-many assumption, pruning technologies and speed. We try to figure out the effects of the proposed approaches. We use two datasets denoted as Verti_Hat and Larva to represent traditional scenario and dense scenario respectively.

#### Scenario Verti_Hat

The results are presented in Table 2. Our tracking method outperforms others in the principal metrics such as OSPA-T, MOTA and IDSW. OSPA-T is a critical metric in the traditional tracking scenario. Our tracking method achieves better performance with near 20% decreasing in OSPA-T compared with the second method, i.e GRASP-MHT. Besides, lower IDSW is a remarkable feature of our method, which can be noticed in the following experiment results as well.

**Table 2:** Performance comparison between our tracking method and others methods for the Verti_Hat dataset.

Method	Traditional Metrics			CLEAR MOT Metrics						
	OSPA-T(↓)	TF(↑)	TCF(↑)	MOTA(↑)	MOTP(↑)	IDSW(↓)	FN(↓)	FP(↓)	REC(↑)	PRC(↑)
MDA	20.4	4.8	0.6	45.3	71.0	514	2334	1094	67.6	81.6
SGTS	20.0	3.5	0.5	46.3	70.9	457	2251	1156	68.7	81.1
Cox’s MHT	21.9	12.2	0.9	50.6	71.4	711	1653	1196	77.0	82.3
GRASP-MHT	18.2	2.2	0.6	52.0	71.2	302	2009	1148	72.1	81.9
Ours	14.7	2.1	0.8	56.9	71.3	68	2026	1011	71.9	83.7

#### Scenario Larva

Larva tracking is a difficult problem with dense object in the clutter background. We collected three sequences with different qualities of image to represent different tracking conditions.

The final row in Table 3 is the weighted average of metrics from three sequences, where for most metrics the weight is the corresponding frame number divided by the total frame number. As for IDSW, FN and FP, we use the sum of three sequences. For this scenario, our method outperforms other methods and ranks first in 6 of total 10 metrics. The recall figure of our method is at an ordinary level, since we try lots of efforts to reduce the number of hypotheses, which may restrict part of correct hypothesis.

**Table 3:** Performance comparison between our tracking method and others methods for the Larva dataset.

Dataset	Method	Traditional Metrics			CLEAR MOT Metrics						
		OSPA-T(↓)	TF(↑)	TCF(↑)	MOTA(↑)	MOTP(↑)	IDSW(↓)	FN(↓)	FP(↓)	REC(↑)	PRC(↑)
**Larva_s1** (typical scene)	MDA	17.2	2.2	0.6	56.7	86	431	2094	1035	74.5	85.6
	SGTS	17.3	1.9	0.6	56.1	85.7	533	1657	1422	79.9	82.2
	Cox’s MHT	15.7	3.2	0.8	48.8	87.1	282	1080	2847	86.9	71.5
	GRASP-MHT	17.6	1.7	0.6	57.3	85.6	425	1641	1450	80.1	82.0
	Ours	13.4	1.2	0.7	67.6	90.0	44	1939	680	76.4	90.2
**Larva_s2** (higher density)	MDA	17.2	1.8	0.7	37.5	86.4	251	1098	1244	73.5	71.0
	SGTS	17.7	1.4	0.6	31.5	85.4	280	946	1614	77.2	66.5
	Cox’s MHT	16.8	2.4	0.9	28.5	88.6	164	478	2331	88.5	61.2
	GRASP-MHT	17.3	1.3	0.7	33.5	85.3	243	860	1657	79.3	66.5
	Ours	12.7	1.0	0.8	57.0	93.0	16	943	831	77.3	79.5
**Larva_s3** (focus drift)	MDA	19.1	1.9	0.5	42.1	85.1	432	5193	1283	56.5	84.0
	SGTS	19.1	1.7	0.5	42.1	84.6	500	4825	1589	59.6	81.7
	Cox’s MHT	17.4	2.2	0.7	46.4	86.8	313	3763	2323	68.5	77.9
	GRASP-MHT	18.8	1.3	0.5	43.2	84.5	393	4750	1638	60.2	81.4
	Ours	17.6	1.1	0.6	50.6	89.3	52	4790	1051	59.9	87.2
**Larva** (average)	MDA	18	2	0.5	47.7	85.7	1114	8385	3562	66.6	82.8
	SGTS	18.1	1.8	0.5	46.6	85.2	1313	7428	4625	70.8	79.7
	Cox’s MHT	16.6	2.7	0.7	44.9	87.2	759	5321	7501	79.2	72.8
	GRASP-MHT	18.1	1.5	0.5	47.9	85.1	1061	7251	4745	71.5	79.5
	Ours	15.1	1.2	0.6	58.8	90.1	112	7672	2562	69.5	87.4

**Fig 8:**
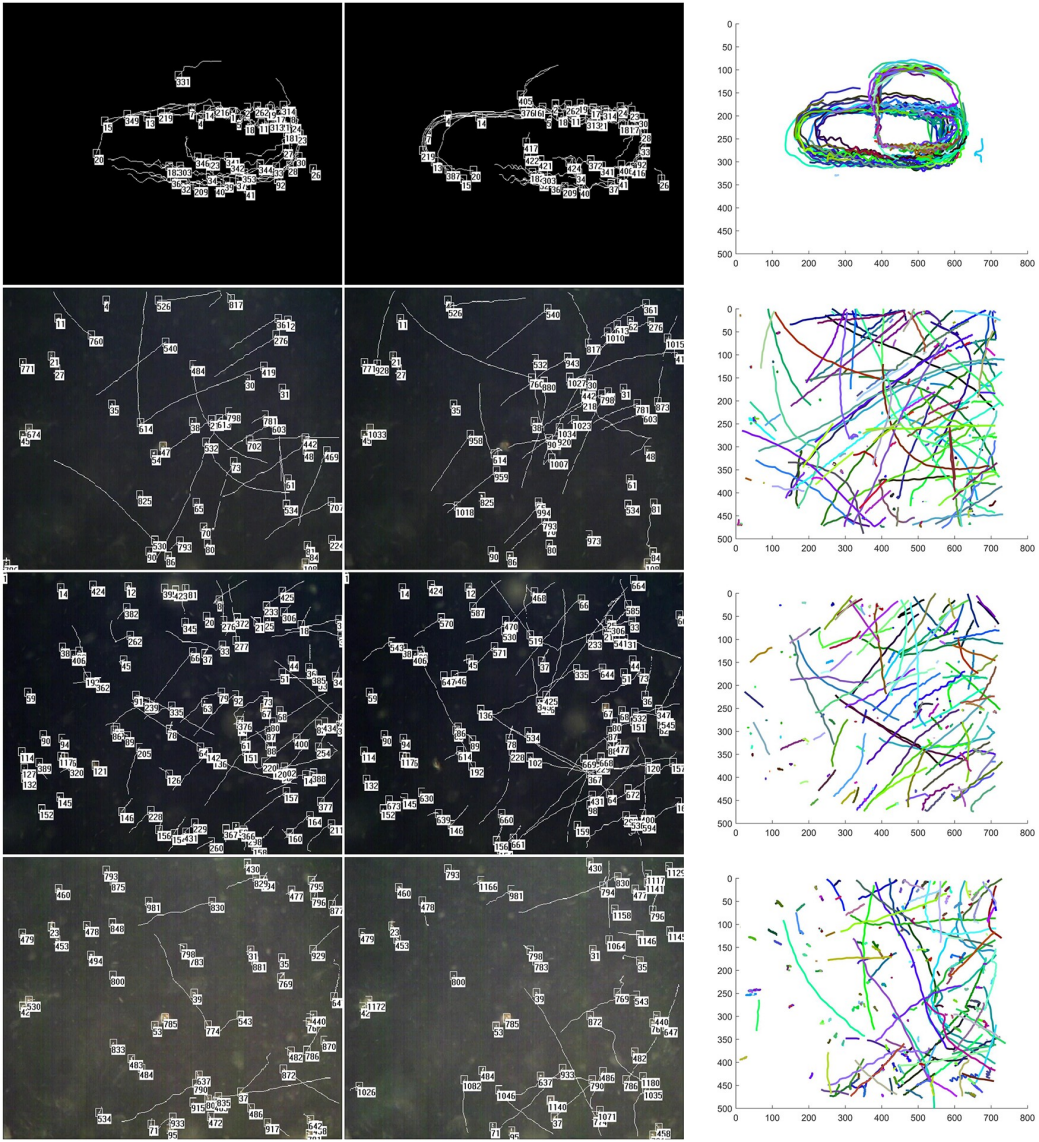
Results on the datasets Larva and Verti_Hat. Each line of the figure shows the tracking results in a sequence clip, including two frame images(last frame and twentieth from the end) and a global projection graphic displaying all trajectories. From top to bottom, four lines of pictures indicate the Verti_Hat,Larva_s1,Larva_s2 and Larva_s3 respectively.

We present part of trajectories in Fig 8. Each line of the pictures shows the tracking results in a sequence clip, including two frame images(last frame and twentieth from the end) and a global projection graphic displaying all trajectories. Object ID is marked on the image. Only last 30 frames of trajectories are drawn to keep the picture more readable. From top to bottom, four lines of pictures indicate the Verti_Hat,Larva_s1,Larva_s2 and Larva_s3 respectively.

#### Impact of one-to-many assumption

By using different constraints for track selection, we also provide an experiment to test the effect of one-to-many assumption. The results of flexible association (one-to-many) and hard association (one-to-one) are listed as Table 4. One-to-many constraint boosts the performance notably according to some critical metrics, where OSPA-T decreases by 22% and MOTA increases by more than 20%. Meanwhile, some metrics appear moderate degeneration such as IDSW because of descent recall. Even though, the one-to-many constraint is effective according to the obvious improvement on critical metrics.

**Table 4:** Performance comparison between our method and its variation under one-to-one constraint for the Larva_s2 dataset.

Method	OSPA-T(↓)	MOTA(↑)	MOTP(↑)	REC(↑)	PRC(↑)
one-to-many	12.7	57.0	93.0	77.3	79.5
one-to-one	16.4	46.1	93.3	58.0	83.1

One-to-one association may refuse some correct hypotheses and produce worse performance in dense situations. In the complex dense scenes, some strong hypotheses encounter long occlusion or merged detection as in Fig 5, where competition for detection occurs. One-to-one association will reject a strong hypothesis that loses in competition while the major part of this hypothesis is correct. Usually, if a tracker could not track an object with consistent ID, it may still produce some small fragments and redeem part of trajectories. Unfortunately, we already removed those weak and short hypotheses at first to keep the number of hypotheses in a tractable scale. Thus, performance will degenerate if we reject objects with small flaws. In comparison, with a looser constraint one-to-many association could preserve the practicable strong hypothesis.

We only provide moderate relaxation for one-to-many constraint. Only a limited number of sharing detections is permitted.

#### Impact of pruning

**Fig 9:**
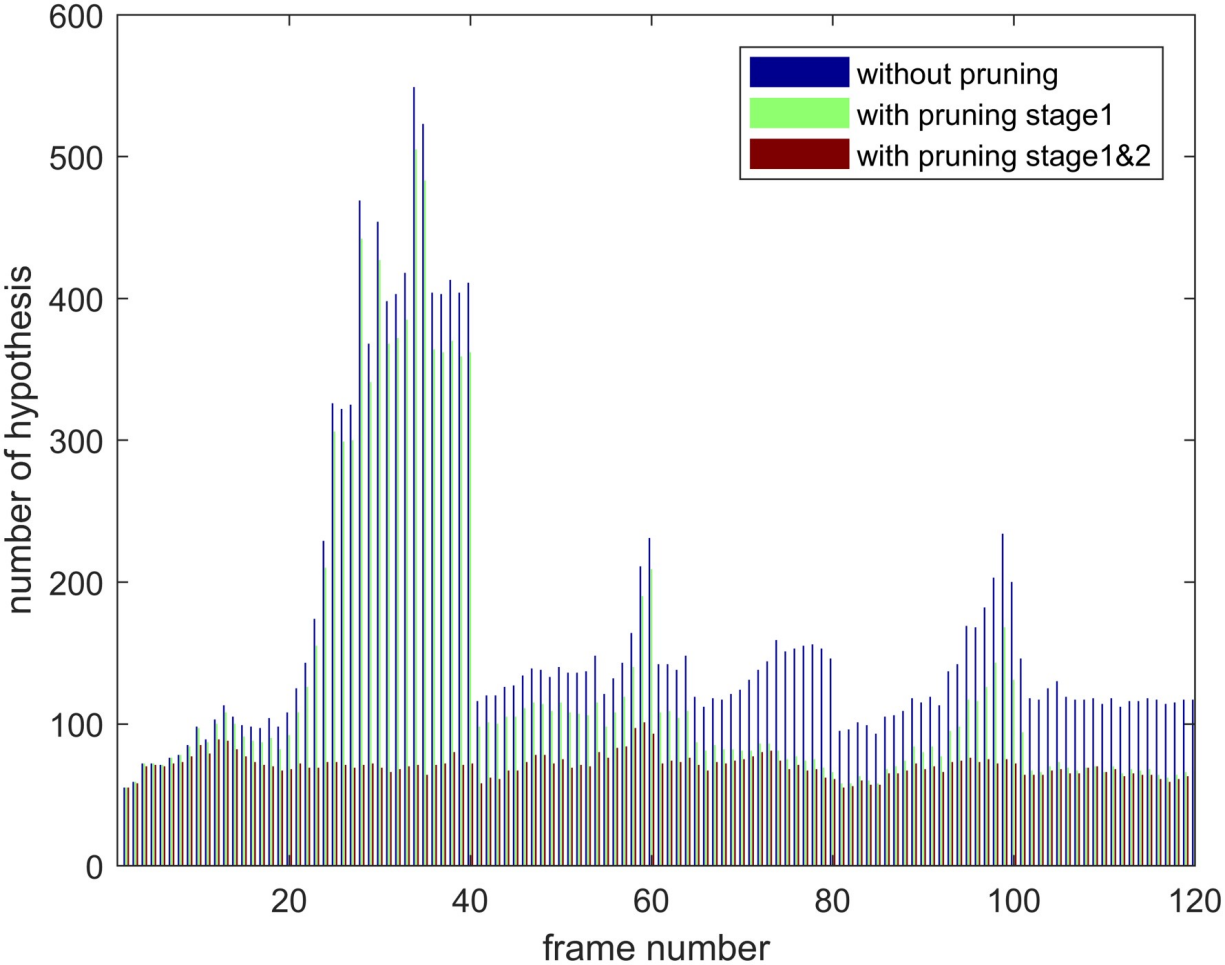
Hypothesis number per time step for three pruning profiles on Verti_Hat.

We evaluate the impact of the proposed pruning technologies on video Verti_Hat by comparing the number of hypothesis per time step for three profiles, namely removing all pruning stages, using only stage 1 and using all pruning stages. As Fig 9 shows, the proposed pruning technologies(stage 1 and 2) significantly reduce the number of hypotheses. The result without any pruning has much higher and unstable hypothesis number, which sometimes rises to near 500 and certainly requires more computations. We also find that stage 2 (loop pruning) produces a better effect than stage 1 by pruning much more hypotheses, which indicates that loop detection based pruning has a significant impact in computation reduction.

#### Analysis of parameters

**Fig 10:**
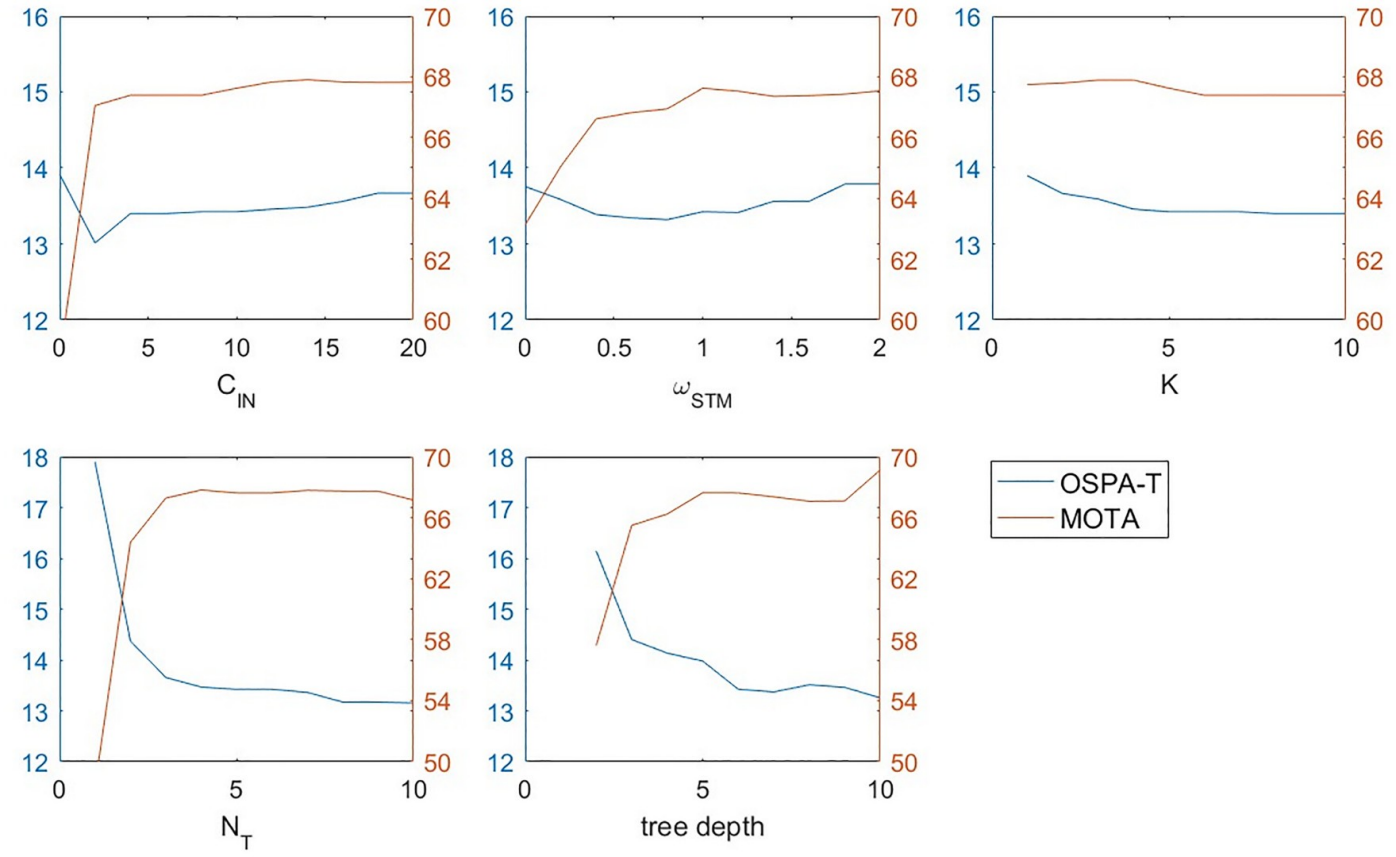
Sensitivity analysis for parameters on Larva_s1.

To deal with the complicated tracking problem, we pre-define many parameters in our algorithm, which produces some difficulties to understand the characteristics of the proposed method. We evaluate the effect and sensibility of different parameters via the experiment presented in Fig 10. We only test on the parameters introduced by ourself. With respect to traditional MHT parameters like *P*_*D*_, λ_*fa*_, λ_*nt*_, we use default ones as in [9, 10]. Since we set *ω*_*STM*_ + *ω*_*LTM*_ = 2, the result for *ω*_*STM*_ also indicates *ω*_*LTM*_’s. We assess different parameters in terms of the MOTA score, OSPA-T.

As in Fig 10, our parameter setting(*ω*_*STM*_ = 1, *ω*_*LTM*_ = 1, *K* = 5, *C*_*IN*_ = 1, *N*_*T*_ = 5) basically ensure both high MOTA score and low OSPA-T, except for tree depth, where deeper tree produces better result. We choose 6 as the depth since deeper tree also requires more computation and memory. The results also show that our algorithm is less sensitive to the most parameter changes. For those parameters with less sensitivity(like *K*, *N*_*T*_), it’s not necessary to precisely locate the highest or lowest point in the flat curve, we can roughly choose a value and the result is still totally comparable. Actually our method is more robust for less dependency on parameter setting.

#### Runtime comparison

Our tracking method achieves comparable performance while maintaining a considerable speed advantage. Cox’s MHT algorithm possesses both well tracking capability and efficient implementation according to the study of Chanho Kim *et.al* [33], so we use it for our speed test. As the Table 5 shows, our tracking method is more than ten times faster than Cox’s MHT. The platform we used is a PC with E5(3.3GHz) CPU. The two methods listed in the table are implemented by the same language(C++) for a fair comparison. The fast implementation of our method is achieved based on the following factors: We screen out weak hypothesis in due course and preserve strong ones; Meanwhile, we employ batch optimization instead of compatibility checking frame by frame.

**Table 5:** Consuming time(second) comparison between our tracking method and others methods for the Larva dataset.

Method	Larva_s1	Larva_s2	Larva_s3	Sum
Cox’s	125.8	351.5	54.2	531.5
Ours	5.8	6.5	4.5	17.9

## Conclusion

In the paper, we propose and implement the methods for an entire tracking processing including detection and tracking for dim small object, from the raw image sequence to identified object trajectories. Firstly, we present MALC to handle merged detection and improved the performance of our tracking method.

Then, we revise multi-hypothesis tracking and propose some technologies for dense small object tracking. We utilize two-stage hypothesis pruning and modified motion score for hypothesis management and generation, which could improve the quality of hypothesis. For the global hypothesis selection, we introduce an extended 0-1 programming with one-to-many association which could tackle severe occlusion. Experimental results are presented to verify the strength of our methods for dense small object, which indicates speed and performance advantages of our tracker.

We will improve our method in the future work by the following respects. Our work contains a series of detection and tracking problems. Many parameters are presented, which will limit the application of the proposed method. We will try to employ less necessary parameters in the improved work. Meanwhile, we utilize a conventional two-stage strategy where detecting is performed before tracking. Actually, detection processing may be integrated into tracking so multi-hypotheses tracking can utilize some medial information from detection.

## Supporting information

S1 Appendix Performance metrics. We introduce all performance metrics used for detection and tracking evaluation in this appendix.
(PDF)
