# Supplementary material for: Multi-appearance segmentation and extended 0-1 programming for dense small object tracking
Source: PLoS One. 2018 Oct 31;13(10):e0206168. doi: 10.1371/journal.pone.0206168 (PMC6209235; doi:10.1371/journal.pone.0206168)
Supplement: S1 Appendix — We introduce all performance metrics used for detection and tracking evaluation in this appendix. (PDF) [file pone.0206168.s001.pdf]

S1 Appendix

Performance metrics

We employ various metrics to evaluate the performance of our detection and tracking method. To evaluate tracking methods more comprehensive and representative, except some traditional metrics like OSPA-T [1] we also utilize some metrics from the field of visual multi-object tracking such as IDSW,MOTA [2]. We introduce all performance metrics in this section including detection and tracking metric.

Detection Metrics

Detection Rate(DR↑) We use the definition that $DR = NC/NT$, where NC and NT are the number of correctly detected objects and true objects respectively.

False Alarms(FA↓) We have $FA = NIC/N$ while NIC is the number of incorrectly detected objects. Meanwhile, N is the length of the sequence.

Standard Deviation of Detection Rate(DR-STD↓) Lower standard deviation of detection rate indicates better stability of detection method.

Tracking Metrics (Traditional)

Traditional metric treat object as point when evaluating tracking performance.

Optimal Sub-pattern Assignment Distance(OSPA-T↓) The modified Optimal Sub-pattern Assignment Distance proposed by Branko Ristic is widely used as measure index in multi-object tracking. c and ℓ are set to 25 as default, and p is 2.

Track Completeness Factor(TCF↑) Track Completeness Factor measures how well we detect a given object after the association [3]. *tol* used in **TCF** is set as 15.

Track Fragmentation(TF↑) Track Fragmentation measures how well we maintain identity [3]. *tol* used in **TF** is set as 15 too.

Tracking Metrics (CLEAT_MOT)

We also used some metrics based on the reference from visual multi-object tracking, which attracts great attention and develops plenty of sophisticated evaluation mechanisms. The representative one is CLEAR_MOT metric [2], which contains MOTA,MOTP, etc. XXX Those metrics were designed for object with a certain size and detection box, rather than a small object with few pixels. However, the small objects in our dense tracking scenario show up more than just few pixels and occupy considerable space(they are still small object with not more than one hundred pixels). Higher density actually exaggerates the effect of their size. Those metrics were designed to evaluate the complex scenario with massive occlusions, which is more complicated than traditional

scenario. Applying those new performance metrics could augment diversity of our result. Under such consideration, we utilized the CLEAR MOT metrics [2].

Number of Identity Switch(IDSW↓) Identity Switch counts the number of emergences when a ground truth target i is matched to hypothesis j and the last known assignment was $k(k \neq j)$ [2].

Multiple Object Tracking Accuracy(MOTA↑) Thanks to its expressiveness, the Multiple Object Tracking Accuracy [2] may be the most widely used figure in evaluating a tracker's performance. The definition of Multiple Object Tracking Accuracy is as 1:

$$MOTA = 1 - \frac{\sum_t (FN_t + FP_t + IDSW_t)}{\sum_t GT_t} \quad (1)$$

It combines three different sources of errors, where t is the index of frame and GT is the number of ground truths. FN is the number of false negatives, and FP is the number of false positives.

Multiple Object Tracking Precision(MOTP↑) The Multiple Object Tracking Precision is the average dissimilarity between all true positives and their corresponding ground truth targets [2].

Ratio Misses Over Total Number(FN↓) The ratio misses in the sequences over the total number of objects presenting in all frames [3].

Ratio False Positive Over Total Number(FP↓) The ratio False Positives over the total number of objects presenting in all frames [3].

Recall(REC↑) The number of correctly matched detections divided by the total number of detections in ground truth.

Precision(PRE↑) The number of correctly matched detections divided by the total number of output detections.

We use up arrow ↑ to represent that higher score indicates better result.The opposite of that, down arrow ↓, means preference to lower score.

References

1. Ristic B, Vo BN, Clark D, Vo BT. A Metric for Performance Evaluation of Multi-Target Tracking Algorithms. IEEE Transactions on Signal Processing. 2011;59(7):3452–3457. doi:10.1109/TSP.2011.2140111.

2. Leal-Taixé L, Milan A, Reid I, Roth S, Schindler K. MOTChallenge 2015: Towards a Benchmark for Multi-Target Tracking. arXiv:150401942 [cs]. 2015;.

3. Perera AGA, Srinivas C, Hoogs A, Brooksby G, Hu W. Multi-Object Tracking Through Simultaneous Long Occlusions and Split-Merge Conditions. In: 2006 IEEE Computer Society Conference on Computer Vision and Pattern Recognition (CVPR'06). vol. 1; 2006. p. 666–673.
